# PEX19 Coordinates Neutral Lipid Storage in Cells in a Peroxisome-Independent Fashion

**DOI:** 10.3389/fcell.2022.859052

**Published:** 2022-04-26

**Authors:** Sven Lyschik, Anna A. Lauer, Tanja Roth, Daniel Janitschke, Markus Hollander, Thorsten Will, Tobias Hartmann, Ron R. Kopito, Volkhard Helms, Marcus O. W. Grimm, Bianca Schrul

**Affiliations:** ^1^ Medical Biochemistry and Molecular Biology, Center for Molecular Signaling (PZMS), Faculty of Medicine, Saarland University, Homburg, Germany; ^2^ Experimental Neurology, Saarland University, Homburg, Germany; ^3^ Center for Bioinformatics, Saarland University, Saarbruecken, Germany; ^4^ Deutsches Institut für Demenzprävention, Saarland University, Homburg, Germany; ^5^ Department of Biology, Stanford University, Stanford, CA, United States; ^6^ Nutrition Therapy and Counseling, Campus Rheinland, SRH University of Applied Health Sciences, Leverkusen, Germany

**Keywords:** lipid droplet, lipid metabolism, peroxisome, endoplasmic reticulum, organelle communication, quantitative SILAC proteomics, lipidomics, protein targeting

## Abstract

Cellular lipid metabolism is tightly regulated and requires a sophisticated interplay of multiple subcellular organelles to adapt to changing nutrient supply. PEX19 was originally described as an essential peroxisome biogenesis factor that selectively targets membrane proteins to peroxisomes. Metabolic aberrations that were associated with compromised PEX19 functions, were solely attributed to the absence of peroxisomes, which is also considered the underlying cause for Zellweger Spectrum Disorders. More recently, however, it was shown that PEX19 also mediates the targeting of the VCP/P97-recuitment factor UBXD8 to the ER from where it partitions to lipid droplets (LDs) but the physiological consequences remained elusive. Here, we addressed the intriguing possibility that PEX19 coordinates the functions of the major cellular sites of lipid metabolism. We exploited the farnesylation of PEX19 and deciphered the organelle-specific functions of PEX19 using systems level approaches. Non-farnesylated PEX19 is sufficient to fully restore the metabolic activity of peroxisomes, while farnesylated PEX19 controls lipid metabolism by a peroxisome-independent mechanism that can be attributed to sorting a specific protein subset to LDs. In the absence of this PEX19-dependent LD proteome, cells accumulate excess triacylglycerols and fail to fully deplete their neutral lipid stores under catabolic conditions, highlighting a hitherto unrecognized function of PEX19 in controlling neutral lipid storage and LD dynamics.

## Introduction

**FIGURE 1:**
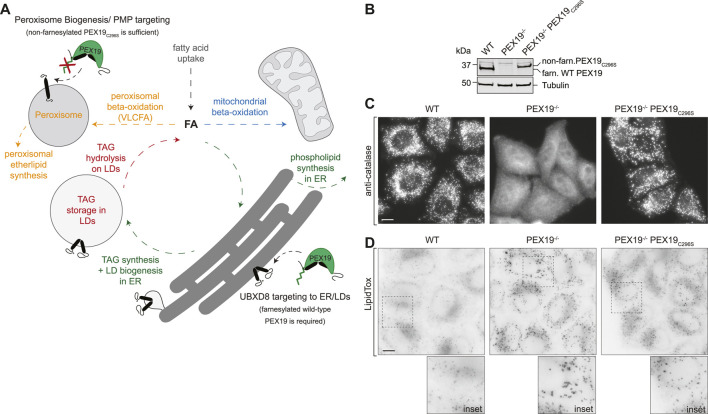
PEX19-compromised cells accumulate excess LDs. **(A)** Schematic illustrating selected lipid metabolic pathways of peroxisomes, LDs, the ER and mitochondria. The role of PEX19 in peroxisome biogenesis and targeting of peroxisomal membrane proteins (PMPs) to peroxisomes as well as in ER/LD targeting of UBXD8 is indicated. For details see text. FA, fatty acid; TAG, Triacylglycerols, VLCFA, very long chain fatty acids **(B)** Western blot analysis of cell lysates from WT, PEX19^−/−^ and PEX19^−/−^PEX19_C296S_ cells as indicated using PEX19-specific antibodies. Farnesylated (farn.) WT PEX19 and non-farnesylated (non-farn.) PEX19_C296S_ are indicated. Note the farnesylation-dependent shift in the electrophoretic mobility of PEX19, confirming that exclusively non-farnesylated PEX19_C296S_ is expressed in PEX19^−/−^PEX19_C296S_ cells. Western blots using anti-tubulin antibodies serve as loading control. **(C)** Stable expression of non-farnesylated PEX19_C296S_ in PEX19 knock-out cells rescues the biogenesis of catalase-positive peroxisomes: Immunofluorescence staining of WT, PEX19^−/−^ and PEX19^−/−^PEX19_C296S_ cells as indicated using anti-catalase antibodies. Maximum intensity projections of z-stacks are shown. Scale bar: 10 µm **(D)** PEX19-compromised cells accumulate excess LDs: Micrographs showing LipidTox-staining of living WT, PEX19^−/−^ and PEX19^−/−^PEX19_C296S_ cells as indicated. Inverted, maximum intensity projections of z-stacks are shown. Scale bar: 10 µm.

Cellular energy metabolism needs to be tightly regulated in order to balance energy production and expenditure, especially in cases where cells need to adapt to changing environmental conditions such as nutrient supply. This dynamic control relies on a sophisticated interplay of multiple subcellular organelles. For instance, when excess fatty acids are taken up into the cell under anabolic conditions, enzymes in the endoplasmic reticulum (ER) catalyze the synthesis of neutral lipids such as triacylglycerols (TAGs), which triggers the formation of lipid droplets (LDs) (Figure 1A). LDs are specialized lipid storage organelles that originate *de novo* from the ER membrane and consist of a hydrophobic neutral lipid core that is encapsulated by a phospholipid monolayer. Although the molecular mechanisms underlying LD biogenesis remain elusive, the current model posits that the local accumulation of TAGs within the phospholipid bilayer of the ER eventually leads to the vectorial budding of a LD from the outer leaflet of the ER bilayer (Renne et al., 2020). Under catabolic conditions, the neutral lipids are hydrolyzed by LD-resident lipases and the resulting fatty acids are transferred to mitochondria where they are broken down via beta-oxidation and used for ATP production. Very long chain fatty acids (VLCFAs) cannot be directly imported into mitochondria and are first oxidized into medium-chain fatty acids (MCFAs) in peroxisomes before they are transferred to mitochondria. The exchange of metabolites between these distinct organelles is key for maintaining energy homeostasis but also poses a risk to the cell as free fatty acids can induce lipotoxicity. Physical membrane contact sites likely ensure efficient exchange of metabolites between these organelles (Bohnert, 2020).

Peroxisomes are central organelles in lipid metabolism and defects in peroxisome function can lead to a spectrum of severe disorders such as Zellweger syndrome, infantile Refsum disease or neonatal adrenoleukodystrophy (Waterham and Ebberink, 2012). Common hallmarks of these Zellweger spectrum disorders are the accumulation of VLCFAs and branched fatty acids, as well as depletion of ether lipids, especially plasmalogens that are essential for healthy brain and lung function. These aberrations in lipid metabolism could all be traced back to a peroxisomal defect and indeed patients usually carry mutations in at least one peroxin (PEX protein); proteins that are important for correct peroxisome biogenesis or function (Wanders et al., 2015).

PEX3, PEX16, and PEX19 are essential peroxisome biogenesis factors in mammals (Hua and Kim, 2016). However, we have previously discovered that PEX19 is not only a protein targeting factor for peroxisomal proteins but also for a ER/LD-destined protein: UBXD8 requires PEX19 as targeting factor for its correct insertion into the ER membrane from where it partitions to the LD surface (Schrul and Kopito, 2016). PEX19 carries a CAAX-motif at its C-terminus and a cysteine within this motif (C296) can be post-translationally farnesylated. The functional relevance of this PEX19 farnesylation has been controversially discussed (Fransen et al., 2005; Vastiau et al., 2006; Rucktaschel et al., 2009; Emmanouilidis et al., 2017; Schrul and Schliebs, 2018), but evidence suggests that it is dispensable for *de novo* peroxisome biogenesis: While cells that are devoid of PEX19 lack peroxisomes, stable expression of the non-farnesylated PEX19_C296S_ mutant in a PEX19^−/−^ background leads to restoration of peroxisome biogenesis (Vastiau et al., 2006; Schrul and Kopito, 2016). In contrast, the non-farnesylated PEX19_C296S_ mutant is not sufficient for correct ER/LD protein targeting of UBXD8 (Schrul and Kopito, 2016). Hence, PEX19 fulfills -at least-two functions in: 1) peroxisome biogenesis, which is farnesylation-independent, and 2) ER/LD protein targeting of UBXD8, which requires farnesylated wild-type PEX19 (Schrul and Kopito, 2016) (Figure 1A).

LD-localized UBXD8 can affect ATGL-mediated LD turnover (Olzmann et al., 2013) but in the absence of wild-type PEX19, UBXD8 is depleted from the LD surface (Schrul and Kopito, 2016). Therefore, the question arises whether PEX19-mediated protein targeting to ER/LDs plays a role in controlling neutral lipid storage and consumption. Likewise, additional LD proteins, besides UBXD8, may rely on PEX19 for their correct subcellular localization and their identification would provide further insight into the physiological role of PEX19-mediated protein targeting to LDs. Farnesylation-deficient PEX19_C296S_ seems to uncouple PEX19-mediated peroxisome biogenesis from protein targeting to ER/LDs and restores peroxisome biogenesis, whereas ER/LD protein targeting is compromised in cells that exclusively express non-farnesylated PEX19_C296S_. It is, however, unclear whether the peroxisomes that are restored in the PEX19^−/−^PEX19_C296S_ cell line are fully functional and whether certain metabolic changes can be specifically attributed to a peroxisome-independent role of PEX19 in ER/LD protein targeting, which may contribute to additional Zellweger spectrum symptoms in patients with defective PEX19. To this end, here we exploited the farnesylation of PEX19 to decipher organelle-specific functions of PEX19. We systematically characterized lipid metabolic and proteomic changes in PEX19^−/−^ cells (no PEX19: no peroxisomes, compromised LD protein targeting) and PEX19^−/−^PEX19_C296S_ cells (only non-farnesylated PEX19: restored peroxisomes, compromised LD protein targeting) and compared them to wild-type cells (farnesylated PEX19: peroxisomes, correct LD protein targeting).

## Results

### PEX19-Compromised Cells Accumulate Excess Lipid Droplets

To confirm the previously described peroxisome phenotype in the CRISPR/Cas9-edited PEX19^−/−^ and PEX19^−/−^PEX19_C296S_ HeLa cells (Schrul and Kopito, 2016), we first verified the absence of PEX19 in PEX19^−/−^ cells and the stable expression of non-farnesylated PEX19_C296S_ in the PEX19^−/−^PEX19_C296S_ cells by quantitative Western Blotting using PEX19-specific antibodies (Figure 1B). Next, we performed immunofluorescence microscopy using antibodies against catalase, which is the hallmark protein of mature peroxisomes and revealed a punctate staining pattern in wild-type cells (Figure 1C). In PEX19^−/−^ cells, only diffuse and no punctate catalase staining could be detected, confirming that mature peroxisomes are indeed absent in these cells and that catalase accumulates in the cytosol. Stable expression of the non-farnesylated PEX19_C296_ mutant in these cells (PEX19^−/−^PEX19_C296S_) resulted in a punctate catalase-staining comparable to wild-type cells, confirming that the biogenesis of mature peroxisomes is apparently restored.

In order to investigate whether PEX19 is involved in neutral lipid storage, we grew wild-type, PEX19^−/−^, and PEX19^−/−^PEX19_C296S_ cells in standard medium without the addition of exogenous fatty acids and stained them with the neutral lipid dye LipidTox to visualize LDs (Figure 1D). Considerably stronger LipidTox-staining could be detected in PEX19^−/−^ cells as well as in PEX19^−/−^PEX19_C296S_ cells when compared to wild-type cells (Figure 1D). Since expression of non-farnesylated PEX19_C296_ rescues peroxisome biogenesis but does not rescue the LD accumulation phenotype, farnesylated PEX19 might play a specific, peroxisome-independent role in controlling neutral lipid storage and consumption.

### Peroxisomal VLCFA Oxidation and Ether Lipid Synthesis Are Independent of PEX19 Farnesylation

In order to comprehensively assess the metabolic consequences upon PEX19 depletion from cells (no peroxisomes, LD accumulation) as well as after stable expression of the non-farnesylated PEX19_C296S_ mutant in a PEX19 knock-out background (peroxisomes present, LD accumulation), we subjected whole cell lysates from these cells to mass spectrometry-based lipidome analyses and compared them to wild-type cells (Full data set in Supplementary Table S1). When grown in standard medium, out of 170 lipid species measured, 24 species were significantly reduced and 31 species significantly increased in PEX19^−/−^ cells (Supplementary Figure S1A). Interestingly, when comparing the lipid profile of PEX19^−/−^PEX19_C296S_ cells to wild-type cells, fewer lipid species were significantly altered (Supplementary Figure S1B,C). This indicates that the expression of non-farnesylated PEX19 rescues some, but not all, PEX19-dependent alterations in lipid metabolism.

**FIGURE 2:**
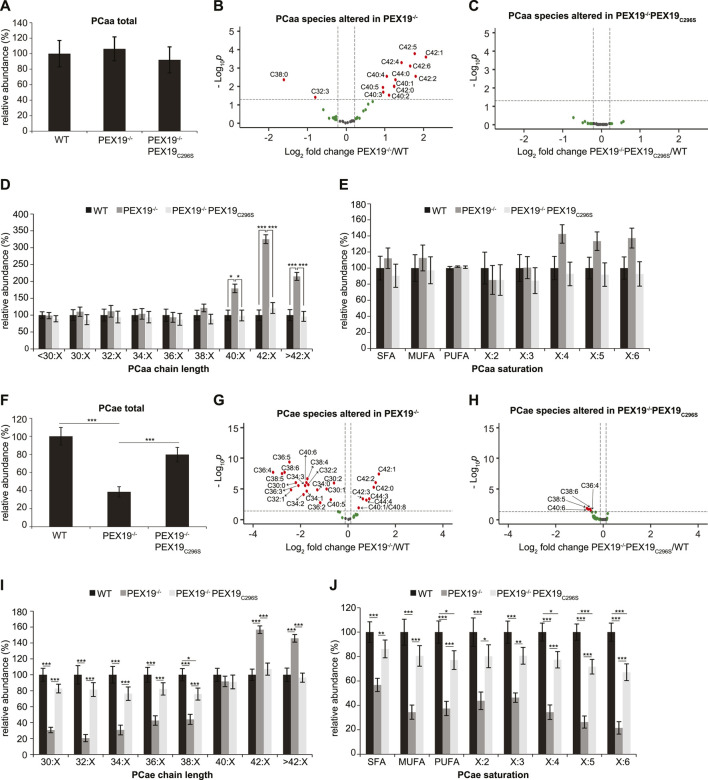
PEX19_C296S_ expression in PEX19 knock-out cells restores peroxisomal VLCFA oxidation and etherlipid synthesis. Shotgun-lipidomics results for ester-linked phosphatidylcholine PCaa **(A–E)** and ether-linked phosphatidylcholine PCae **(F–J)** species. **(A)** Bar diagram showing the relative abundance of total PCaa species in WT, PEX19^−/−^ and PEX19^−/−^PEX19_C296S_ cells. ANOVA revealed no significant changes between the cell lines. **(B,C)** Volcano plots of PCaa subspecies analyses. 43 PCaa species were analyzed and logarithmically plotted against the *p*-value (derived from unpaired, two-sided student’s t-test) after FDR-correction, illustrating changes in PEX19^−/−^ cells **(B)** and PEX19^−/−^PEX19_C296S_ cells **(C)** compared to WT cells, respectively. Grey dots: lipid species without significant changes. Green dots: lipid species with a fold change greater than the average SEM but with a *p*-value >0.05. Red dots: significantly altered (*p*-value <0.05) lipid species with a greater fold change than the average SEM. **(D)** Bar diagram showing the relative abundance of PCaa species categorized by acyl chain length (sum of acyl chains at sn−1 and sn−2 positions). **(E)** Bar diagram showing the relative abundance of PCaa species categorized by unsaturation of the acyl chains (sum of double bonds in acyl chains at sn−1 and sn−2 positions). SFA, saturated fatty acids, MUFA, mono-unsaturated fatty acids, PUFA: poly-unsaturated fatty acids. **(F–J)** Analogous to **(A–E)** but illustrating the analyses for 39 ether-linked PCae species instead of PCaa. Error bars indicate SEM and statistical significance between the groups was determined by an ANOVA followed by Tukey post-hoc test. *,**, *** indicate *p*-values <0.05, <0.01, and <0.001, respectively. Non-significant changes are not indicated. For each cell line 11 individual samples were analyzed with three technical replicates each. See also Supplementary Figure S1.

Peroxisomes are required for the beta-oxidation of VLCFAs and for ether lipid synthesis (see Figure 1A). We aimed to explore whether the exclusive expression of non-farnesylated PEX19_C296S_ in cells results in the full restoration of peroxisomal function. While the total amount of all phosphatidylcholine phospholipid (PCaa) species remained constant when comparing wild-type, PEX19^−/−^ and PEX19^−/−^PEX19_C296S_ cells (Figure 2A), significant changes were detected in the PCaa subspecies composition when PEX19 was absent from cells (Figure 2B). Especially, PCaa species with acyl chain lengths above 40 accumulated in PEX19^−/−^ cells (Figure 2D), which is consistent with the absence of peroxisomes and in turn beta-oxidation activity of VLCFAs in this cell line. VLCFAs are defined as fatty acids with at least 22 carbon atoms. Since PCaa phospholipids contain two acyl chains, a sum of carbon atoms above 40 indicates that at least one acyl chain belongs to the class of VLCFAs.

In accordance with the accumulation of VLCFAs, also polyunsaturated fatty acid (PUFA)- containing PCaa species with four or more double bonds accumulate in PEX19^−/−^ cells, however, without statistical significance and without altering the total amount of PCaa PUFA species (Figure 2E). PCaa species with more than 2 double bonds reflect only a minor fraction of the overall PUFA population (Supplementary Figure S1D). Interestingly, the accumulation of VLCFA PCaa species is completely restored in PEX19^−/−^PEX19_C296S_ cells (Figures 2C–E) indicating that expression of non-farnesylated PEX19 is sufficient to fully restore peroxisomal beta-oxidation activity.

Regarding ether lipid synthesis, total ether-linked phosphatidylcholine (PCae) species were severely depleted in PEX19^−/−^ cells (Figure 2F), which is again consistent with the absence of peroxisomes in this cell line. PCae subspecies analyses revealed that the majority of PCae species was indeed significantly reduced in PEX19^−/−^ cells, whereas 7 PCae species were enriched (Figure 2G). These enriched species all have chain lengths above 42, which is in line with the observation that beta-oxidation of VLCFAs is compromised in the absence of PEX19 and peroxisomes (Figures 2G,I). Depletion of the other PCae species was independent of a particular PCae acyl chain length below 38 or the acyl chain saturation degree (Figures 2I,J). As observed for PCaa, also the effects on ether-linked PCae species were rescued by the expression of non-farnesylated PEX19_C296S_ (Figures 2F,H–J). An incomplete rescue by PEX19_C296S_ could be observed with respect to PCae acyl chain saturation. While saturated fatty acid (SFA) and mono-unsaturated fatty acid (MUFA) PCae species were indistinguishable in wild-type and PEX19^−/−^PEX19_C296S_ cells, PUFA PCae species, especially those with 5 or 6 double bonds, remained about 20% reduced in PEX19^−/−^PEX19_C296S_ cells (Figure 2J). This, however, did not result in an overall reduction of total PCae when comparing wild-type cells and PEX19^−/−^PEX19_C296S_ cells (Figure 2F), presumably because these species resemble only a minor fraction of the total PCae species.

Together, these data indicate that non-farnesylated PEX19_C296S_ restores the biogenesis of catalase-positive peroxisomes in PEX19^−/−^ cells and that these peroxisomes are metabolically fully functional with respect to beta-oxidation and ether lipid synthesis. This provides further evidence that farnesylation of PEX19 is indeed dispensable for correct peroxisome biogenesis and function.

### Triacylglycerols Accumulate in PEX19-Compromised Cells

**FIGURE 3:**
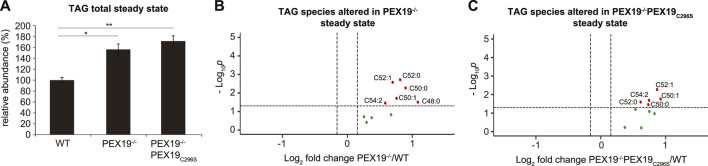
Triacylglycerols accumulate in PEX19-compromised cells. Shotgun-lipidomics results for TAG species. **(A)** Bar diagram showing the relative abundance of total TAG species in WT, PEX19^−/−^ and PEX19^−/−^PEX19_C296S_ cells. Error bars indicate SEM and statistical significance between the groups was determined by an ANOVA followed by Tukey post-hoc test. *,** indicate *p*-values <0.05 and <0.01, respectively. Non-significant changes are not indicated. **(B,C)** Volcano plots of TAG subspecies analyses. 10 TAG species were analyzed and logarithmically plotted against the *p*-value (derived from unpaired, two-sided Student’s t-test) after FDR-correction, illustrating changes in PEX19^−/−^ cells **(B)** and PEX19^−/−^PEX19_C296S_ cells **(C)** compared to WT cells, respectively. Grey dots: lipid species without significant changes. Green dots: lipid species with a fold change greater than the average SEM but with a *p*-value >0.05. Red dots: significantly altered (*p*-value <0.05) lipid species with a greater fold change than the average SEM. For each cell line 11 individual samples were analyzed with three technical replicates each.

In accordance with our observation that PEX19^−/−^ and PEX19^−/−^PEX19_C296S_ cells accumulate excess LDs (Figure 1D), shotgun lipidomics revealed that TAG levels in both cell lines were elevated by approximately 60% compared to wild-type cells (Figure 3A). TAG subspecies analyses show that similar TAG species accumulated in both cell lines (Figures 3B,C). Interestingly, and in contrast to the effects observed for peroxisomal beta-oxidation and ether lipid synthesis, non-farnesylated PEX19 is not sufficient to restore normal TAG abundance in cells, suggesting that this is a PEX19-specific and peroxisome-independent PEX19 phenotype.

### *De Novo* Triacylglycerol Synthesis is Not Compromised in PEX19^−/−^PEX19_C296S_ Cells

**FIGURE 4:**
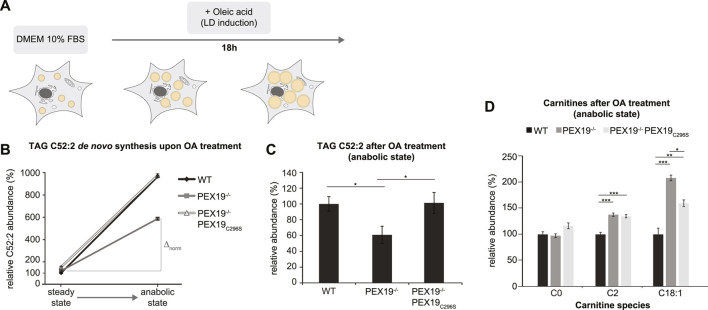
*De novo* TAG synthesis is not compromised in PEX19^−/−^PEX19_C296S_ cells. **(A)** Schematic outline depicting that cells were shifted to anabolic conditions by incubation in DMEM medium containing 10% FBS and oleic acid (OA) for 18 h. **(B,C)** Shotgun-lipidomics results for TAG C52:2 species upon OA treatment. **(B)** Plot indicating TAG *de novo* synthesis upon OA treatment for WT, PEX19^−/−^ and PEX19^−/−^PEX19_C296S_ cells using C52:2 as a metric (Starting points = steady state; endpoints = anabolic state). Values were normalized to 100% in WT cells under steady state conditions before OA treatment. Relative C52:2 *de novo* synthesis as fold change is defined by the ratio between C52:2 levels under anabolic condition and C52:2 levels at steady state before OA treatment and is 9.6 for WT, 4.9 for PEX19^−/−^ and 6.4 for PEX19^−/−^PEX19_C296S_ cells. ∆_norm_ indicates the absolute C52:2 synthesis as defined by the difference of C52:2 levels under anabolic conditions and C52:2 levels at steady state before OA treatment for each cell line and is 864 for WT, 467 for PEX19^−/−^ and 823 for PEX19^−/−^PEX19_C296S_ cells **(C)** Bar diagram showing the relative abundance of TAG C52:2 species accumulating after OA treatment in WT, PEX19^−/−^ and PEX19^−/−^PEX19_C296S_ cells. Values were normalized to 100% in WT cells. **(D)** Bar diagram showing the relative abundance of carnitine species accumulating after oleic acid treatment in WT, PEX19^−/−^ and PEX19^−/−^PEX19_C296S_ cells. Error bars indicate SEM and statistical significance between the groups was determined by an ANOVA followed by Tukey post-hoc test. *,**, *** indicate *p*-values <0.05, <0.01, and <0.001, respectively. Non-significant changes are not indicated. For each cell line 5 individual samples were analyzed with three technical replicates each. See also Supplementary Figure S2.

Excess TAG and LD accumulation in PEX19-compromised cells at steady state could either result from increased lipogenesis or from decreased lipolysis. In order to test whether *de novo* TAG synthesis is altered in the absence of wild-type PEX19, we cultivated wild-type, PEX19^−/−^ and PEX19^−/−^PEX19_C296S_ cells in oleic acid-containing medium overnight (Figure 4A). Shotgun-lipidomics revealed that exogenous oleic acid was predominantly incorporated into TAG C52:2 species (Supplementary Figure S2A), which we therefore selected as a metric for *de novo* TAG synthesis. As expected, the addition of exogenous oleic acid led to the induction of LD biogenesis in cells (Supplementary Figure S3).

While TAG C52:2 levels were about 8.6-fold increased in wild-type cells upon oleic acid treatment, only a 3.9- and 5.4-fold increase was detected in PEX19^−/−^ and PEX19^−/−^PEX19_C296S_ cells, respectively (Figure 4B). This could suggest that *de novo* TAG synthesis may be compromised in both PEX19^−/−^ and PEX19^−/−^PEX19_C296S_ cells. However, both cell lines already had exhibited excess TAG storage in LDs before they were subjected to oleic acid treatment (compare Figures 3, 4B starting point “steady state”), which raises the question of whether a maximum TAG storage capacity in LDs exists in cells. Indeed, similar TAG C52:2 amounts were detected in wild-type and PEX19^−/−^PEX19_C296S_ cells after oleic acid treatment (Figures 4B,C). This indicates that PEX19^−/−^PEX19_C296S_ cells possess full TAG storage capacity, and that the apparent compromise in relative *de novo* TAG synthesis is an indirect effect of reaching maximum storage capacity, while fewer TAG molecules need to be synthesized *de novo* (Figure 4B: ∆_norm_ in PEX19^−/−^PEX19_C296S_ cells is 823 versus ∆_norm_ in wildtype cells is 864).

In contrast, PEX19^−/−^ cells accumulated approximately 40% less TAG C52:2 upon oleic acid treatment (Figure 4C), which indicates that in the absence of PEX19 indeed significantly less TAGs are synthesized (Figure 4B: ∆_norm_ in PEX19^−/−^ cells is 467 vs. ∆_norm_ in wildtype cells is 864) although the maximum storage capacity is not reached. We can exclude an alternative route whereby C18:1 is esterified into other TAG species than C52:2 in PEX19^−/−^ cells since a similar defect in TAG synthesis was observed when quantifying all TAG species in these cells (Supplementary Figures S2B,C). This shows that in the absence of PEX19, and in turn in the absence of peroxisomes, cells produce less TAGs. Expression of non-farnesylated PEX19_C296_, and therefore restoration of peroxisome function, rescues this anabolic defect and TAGs accumulate to the same extent as in wild-type cells.

Compromised TAG *de novo* synthesis in PEX19^−/−^ cells could be due to two mechanisms: First, the exogenous fatty acids may not be taken up efficiently, or second, fatty acids may be shuttled into other metabolic pathways. To differentiate between these two possibilities, we performed lipidomics analyses of carnitine species, which are required for the transport of fatty acids across mitochondrial membranes. This revealed that C2 species, reflecting acetyl-CoA as the end product of mitochondrial beta-oxidation, and C18:1 species, reflecting carnitine-coupled oleic acid that is transported into mitochondria, are significantly elevated in PEX19^−/−^ and PEX19^−/−^PEX19_C296S_ cells, suggesting increased mitochondrial beta-oxidation activity in both cell lines. Interestingly, C18:1 carnitines accumulate significantly more in PEX19^−/−^ cells than in PEX19^−/−^PEX19_C296S_ cells (Figure 4D), which indicates that more exogenous oleic acid is shuttled into mitochondrial beta-oxidation in the absence of peroxisomes.

Taken together, our results indicate that PEX19^−/−^ cells are metabolically distinct from wild-type and PEX19^−/−^PEX19_C296S_ cells as they preferentially shuttle exogenous fatty acids into mitochondrial beta-oxidation and in turn esterify fewer fatty acids into TAGs. As a result, less TAGs are stored in PEX19^−/−^ cells upon addition of exogenous fatty acids. Farnesylation-deficient PEX19_C296S_ cells also esterify fewer fatty acids into TAGs than wild-type cells and shuttle surplus fatty acids into mitochondrial beta-oxidation. This, however, most likely stems from the fact that PEX19^−/−^PEX19_C296S_ cells already exhibit elevated TAG storage compared to wild-type cells under standard growth conditions and, as a consequence, less TAG molecules need to be synthesized in these cells to reach wild-type-like TAG storage capacity. Since wild-type and PEX19_C296_-expressing cells accumulate similar TAG amounts upon oleate-treatment and since both cell lines harbor peroxisomes, we conclude that functional peroxisomes—rather than a specific PEX19 function—are required for normal TAG storage under anabolic conditions.

### Triacylglycerols and Lipid Droplets Cannot Be Fully Depleted in PEX19-Compromised Cells

**FIGURE 5:**
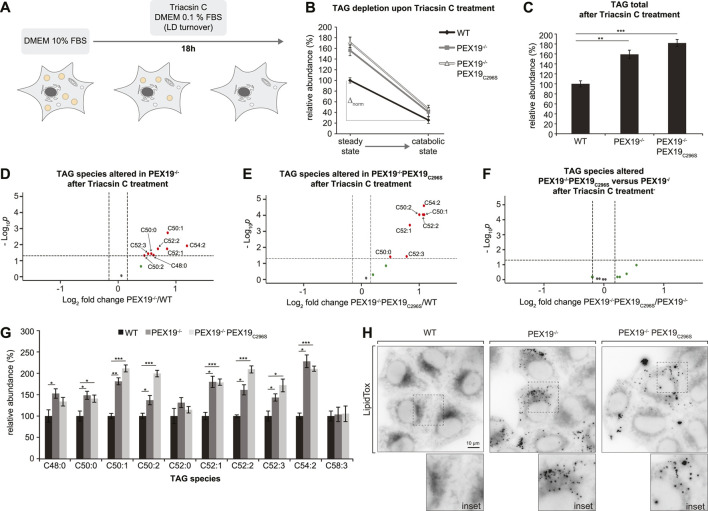
TAGs and LDs cannot be fully depleted in PEX19-compromised cells. **(A)** Schematic outline depicting that cells were shifted to catabolic conditions by incubation in DMEM medium with 0.1% FBS and in presence of Triacsin C for 18 h. **(B–G)** Shotgun-lipidomics results for TAG species upon Triacsin C treatment. **(B)** Plot indicating TAG depletion upon Triacsin C treatment for WT, PEX19^−/−^ and PEX19^−/−^PEX19_C296S_ cells (Starting points = steady state; endpoints = catabolic state). TAG values were normalized to 100% in WT cells under steady state conditions before Triacsin C treatment. Relative TAG depletion upon Triacsin C treatment overnight is 74.4% in WT, 73.9% in PEX19^−/−^ and 72.9% in PEX19^−/−^PEX19_C296S_ cells. ∆_norm_ indicates the absolute TAG depletion as defined by the difference of TAG levels under steady state conditions before Triacsin C treatment and TAG levels at catabolic state for each cell line and is 74 for WT, 116 for PEX19^−/−^ and 125 for PEX19^−/−^PEX19_C296S_ cells. **(C)** Bar diagram showing the relative abundance of total TAG species remaining after Triacsin C treatment in WT, PEX19^−/−^ and PEX19^−/−^PEX19_C296S_ cells. Values were normalized to 100% in WT cells. **(D–F)** Volcano plots of TAG subspecies analyses. 10 TAG species were analyzed and logarithmically plotted against the *p*-value (derived from unpaired, two-sided Student’s t-test) after FDR-correction, illustrating changes in PEX19^−/−^ cells **(D)** and PEX19^−/−^PEX19_C296S_ cells **(E)** compared to WT cells, respectively, as well as changes between PEX19^−/−^ and PEX19^−/−^PEX19_C296S_ cells **(F)**. Grey dots: lipid species without significant changes. Green dots: lipid species with a fold change greater than the average SEM but with a *p*-value >0.05. Red dots: significantly altered (*p*-value <0.05) lipid species with a greater fold change than the average SEM. **(G)** Bar diagram showing the relative abundance of TAG species in WT, PEX19^−/−^ and PEX19^−/−^PEX19_C296S_ cells. Error bars indicate SEM and statistical significance between the groups was determined by an ANOVA followed by Tukey post-hoc test. *,**, *** indicate *p*-values <0.05, <0.01, and <0.001, respectively. Non-significant changes are not indicated. For each cell line 8 individual samples were analyzed with three technical replicates each. **(H)** Micrographs showing LipidTox-staining of living WT, PEX19^−/−^ and PEX19^−/−^PEX19_C296S_ cells as indicated. Inverted, maximum intensity projections of z-stacks are shown. Scale bar: 10 µm.

Since excess TAG and LD accumulation in PEX19^−/−^ and PEX19^−/−^PEX19_C296S_ cells under normal growth conditions (Figures 1D, 3) does not stem from enhanced TAG synthesis (Figure 4), we next tested whether this results from decreased lipolysis (i.e., TAG hydrolysis). Therefore, we subjected the cells to catabolic growth conditions, in which existing LDs are turned over and new LD biogenesis is inhibited (Figure 5A). Cells were grown over night in medium containing 0.1% FBS and in the presence of the long chain fatty acyl CoA synthetase inhibitor Triacsin C. In all three cell lines the total abundance of TAGs decreased by approximately 73% compared to the TAG levels that were measured in these cells under standard growth conditions (Figure 5B). This indicates that Triacsin C treatment abolished the synthesis of new TAGs and led to the depletion of existing TAGs in all cell lines. However, in PEX19^−/−^ and PEX19^−/−^PEX19_C296S_ cells, the absolute TAG levels remaining after the treatment were elevated by approximately 70% compared to wild-type cells (Figures 5B,C), which was also observed under regular growth conditions without Triacsin C treatment (compare Figure 5C with Figure 3A). Therefore, more TAG molecules had been hydrolyzed per time in PEX19^−/−^ and PEX19^−/−^PEX19_C296S_ cells compared to wild-type cells, which indicates that lipolysis *per se* is not compromised in PEX19^−/−^ and PEX19^−/−^PEX19_C296S_ cells (Figure 5B: ∆_norm_ in wild-type cells is 74 versus ∆_norm_ of 116 and 125 in PEX19^−/−^ cells and PEX19^−/−^PEX19_C296S_, respectively). TAG subspecies analyses indicate that similar TAG species accumulated in PEX19^−/−^ and PEX19^−/−^PEX19_C296S_ cells (Figures 5D–G).

In order to distinguish between a potential defect in LD turnover and TAG accumulation within the ER membrane, we stained cells with LipidTox. This revealed that considerably more LDs remained detectable in PEX19^−/−^ and PEX19^−/−^PEX19_C296S_ cells than in wild-type cells (Figure 5H). In summary, cells without wild-type PEX19 are unable to completely deplete their TAG/LD stores. Since PEX19_C296S_ does not rescue this phenotype, we conclude that this effect is unrelated to a general, metabolic peroxisome function. Instead, we postulate that incomplete TAG/LD storage depletion is a specific consequence of compromised PEX19 activity, presumably defective targeting of proteins to ER/LDs.

### Identification of PEX19-Dependent Lipid Droplet Proteins by Quantitative Mass Spectrometry

A range of LD-localized proteins control TAG hydrolysis and therefore LD turnover. The incomplete LD turnover as observed in PEX19^−/−^ and PEX19^−/−^PEX19_C296S_ cells could result from a defect in PEX19-mediated protein targeting to LDs. We previously showed that the farnesylation of PEX19 is required to properly target the protein UBXD8 to the ER from where it partitions to the LD monolayer membrane (Schrul and Kopito, 2016). Solely the absence of UBXD8 from LDs, however, does not explain the LD phenotype that we have observed in this study because it was shown previously that LD-localized UBXD8 stabilizes LDs by preventing ATGL-mediated lipolysis (Olzmann et al., 2013). In contrast, here we observed that the lack of PEX19 farnesylation, and in turn decreased UBXD8 levels on LDs, also leads to accumulation of TAGs and LDs. This suggests that PEX19 plays a more global role in controlling TAG storage and consumption, potentially by controlling the LD proteome on a broader scale that is not limited to UBXD8.

**FIGURE 6:**
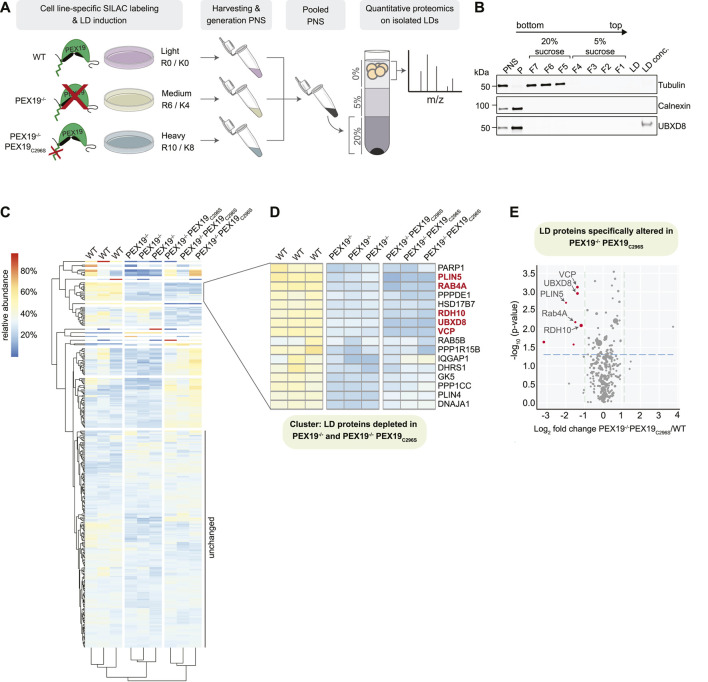
Identification of PEX19-dependent LD proteins by quantitative mass spectrometry. **(A)** Schematic outline of stable isotope labeling of wild-type (light: R0/K0), PEX19^−/−^ (medium: R6/K4) and PEX19^−/−^PEX19_C296S_ (heavy: R10/K8) cells and LD isolation. LDs were isolated from pooled post-nuclear supernatants by density gradient centrifugation. Buoyant LD fractions were further processed for quantitative mass spectrometry. **(B)** Representative Western Blot analysis of samples taken during the LD purification procedure from wild-type cells. PNS, post-nuclear supernatant, P, pellet, F7-F5, fractions from step gradient containing 20% sucrose, F4-F1, fractions from step gradient containing 5% sucrose, LD, aliquot from LD fraction, LD conc., proteins in the LD fraction were concentrated by TCA precipitation. Antibodies against tubulin and calnexin define cytosol-containing and ER-containing fractions, respectively. Anti-UBXD8 antibodies detect endogenous UBXD8 in the PNS input, in the ER-membrane-containing pellet (P) and on LDs. **(C)** Heat map showing 2-way hierarchical clustering of SILAC-proteomics results for three independent experiments derived from wild-type, PEX19^−/−^ and PEX19^−/−^PEX19_C296S_ cells as indicated. Relative abundances are indicated according to the color code shown on the upper left. Proteins that are equally abundant on LDs in all three cells lines have a theoretical value of 33.3%. Red color indicates proteins that are relatively enriched, dark blue color indicates proteins that are relatively depleted. **(D)** Subcluster from heat map in **(C)** composed of LD proteins that are less abundant in LD fractions derived from PEX19^−/−^ and PEX19^−/−^PEX19_C296S_ cells compared to wild-type cells. Proteins that are depleted from LDs in PEX19^−/−^PEX19_C296S_ cells compared to wild-type cells with statistical significance (compare volcano plot in **(E)**) are indicated in red. **(E)** Volcano plot illustrating differences in the LD proteome of PEX19^−/−^PEX19_C296S_ cells compared to wild-type cells. Proteins that are significantly (*p* ≤ 0.05) depleted from LDs in PEX19^−/−^PEX19_C296S_ cells are indicated by red dots whereas the dot diameter reflects overall abundance of respective peptides identified in the pooled SILAC sample. 95% confidence intervals are indicated by vertical green-dotted lines. Horizontal, blue-dotted line indicates a *p*-value of 0.05.

In order to identify the PEX19-dependent LD proteome, we aimed to determine the relative abundance of LD-localized proteins in wild-type, PEX19^−/−^ and PEX19^−/−^PEX19_C296S_ cells, respectively, by quantitative SILAC (stable isotope labeling with amino acids in cells)-based proteomics (Figure 6A). Therefore, the three cell lines were individually grown in medium containing either light, medium or heavy isotope labeled amino acids. After induction of LD formation by oleic acid, post-nuclear supernatants from each cell line were pooled and LDs isolated by density gradient fractionation. The quality of LD-enriched buoyant fractions was assessed by quantitative Western Blotting, which revealed that these were virtually free of ER membranes and cytosolic proteins as judged by the absence of the marker proteins calnexin and tubulin, respectively (Figure 6B). UBXD8 was detected in the pellet fraction, containing ER-membranes as well as in the LD fraction, consistent with its dual localization to the ER and LDs. Liquid chromatography—tandem mass spectrometry was employed to determine the identity as well as the relative abundance of light- (wild-type-derived), medium-(PEX19^−/−^-derived) and heavy- (PEX19^−/−^PEX19_C296S_-derived) labeled proteins in the LD fraction. We reasoned that proteins, which are depleted from LDs in both, PEX19^−/−^ and PEX19^−/−^PEX19_C296S_ cells, specifically depend on PEX19-mediated protein targeting rather than being indirectly affected in their subcellular localization by peroxisomal defects.

309 proteins were identified in the LD fractions from three independent experiments with at least 2 unique peptides and 5 peptide spectrum matches (PSMs) (full data in Supplementary Table S2). Most of these were similarly abundant in all three cell lines (cluster “unchanged” in Figure 6C and Supplementary Table S2). Among these were any known lipid-metabolic enzymes that are required for TAG synthesis and hydrolysis including ATGL, which is the rate-limiting and major TAG lipase on LDs, as well as its activator CGI-58 (Lass et al., 2011). In contrast, the abundance of several proteins was altered in LD fractions from PEX19^−/−^ only but not from PEX19^−/−^PEX19_C296S_ cells, suggesting that the lack of peroxisomes broadly affects protein recruitment to LDs or, alternatively, leads to unspecific enrichment of peroxisome-destined proteins in the LD fraction. For example, the peroxisomal Alkyl-DHAP synthase AGPS, which is important for etherlipid synthesis, was highly enriched in LD fractions from PEX19^−/−^ cells, but its abundance was restored to wild-type levels in PEX19^−/−^PEX19_C296S_ cells, in which functional peroxisomes are restored.

Proteins that were depleted from LDs in both PEX19^−/−^ and PEX19^−/−^PEX19_C296S_ cells were of particular interest as they represent the LD proteome that specifically depends PEX19-mediated LD targeting and not just on PEX19, and therefore simply the presence of peroxisomes in general (cluster in Figure 6D). Of these, only 5 proteins were significantly depleted from LDs with a confidence greater than 95% when comparing PEX19^−/−^PEX19_C296S_ with wild-type cells (Figure 6E): UBXD8, VCP, PLIN5, Rab4A, and RDH10.

UBXD8 was previously identified as a PEX19-depended LD protein and therefore served as a positive control. Consistent with previous quantifications *via* quantitative Western blotting (Schrul and Kopito, 2016), UBXD8 abundance on LDs was decreased to approximately 30%. VCP is a known interaction partner of UBXD8 and it relies on UBXD8 for its recruitment to the LD surface (Olzmann et al., 2013). Consistently, VCP was stoichiometrically depleted from LDs as well (Figure 6E).

PLIN5, Rab4A and RDH10 were all depleted from LDs to a degree comparable to or even stronger than UBXD8 and VCP in PEX19^−/−^PEX19_C296S_ cells (Figure 6E). All three proteins have previously been characterized as LD-localized proteins: PLIN5 belongs to the perilipin family of LD proteins with multiple roles in lipid metabolism, including the control of TAG storage and mediating physical contacts between LDs and mitochondria (Mason and Watt, 2015; Mass Sanchez et al., 2021). RDH10 is a retinol dehydrogenase with important functions in retinol metabolism and, similar to UBXD8, dually localizes to the ER as well as to LDs (Jiang and Napoli, 2013). Dynamic redistribution of RDH10 between the ER and LDs during metabolic remodeling has recently been discussed (Klyuyeva et al., 2021). Rab4A is a GTPase, which also localizes to endosomal compartments and is implicated in GLUT4 trafficking at the plasma membrane. Its function on LDs is currently unknown but RNAi-mediated knock-down of Rab4A leads to the accumulation and clustering of LDs in macrophages (Mejhert et al., 2020). For all these proteins it was unknown how they are targeted to LDs. Our results indicate that they rely on the presence of wild-type, farnesylated PEX19 for their correct LD localization and, in analogy to UBXD8, presumably rely on PEX19-mediated organelle targeting. Western Blot analyses of cell lysates revealed that UBXD8 is equally abundant in wild-type, PEX19^−/−^ and PEX19^−/−^PEX19_C296S_ cells (Supplementary Figure S3E) albeit being significantly depleted from LDs in PEX19^−/−^ and PEX19^−/−^PEX19_C296S_ (Figure 6). This is consistent with our previous findings that in the absence of wild-type Pex19, UBXD8 is mistargeted to mitochondria, from where it cannot partition to LDs but where it is apparently protected from proteasomal degradation and accumulates (Schrul and Kopito, 2016). In contrast, Rab4A and RDH10 are less abundant in lysates from PEX19^−/−^ and PEX19^−/−^PEX19_C296S_ cells (Supplementary Figure S3E) and are depleted from LDs derived from these cell lines (Figure 6 and Supplementary Figure S3D). These observations are consistent with a model in which Rab4A and RDH10 are, like UBXD8, also clients for PEX19-mediated ER targeting but are turned over when their correct membrane insertion fails. We also attempted to quantify the abundance of PLIN5 in lysates derived from the three cell lines in order to determine whether it would, in the absence of farnesylated PEX19, accumulate as observed for UBXD8 or whether it would be degraded as observed for Rab4A and RDH10. We could, however, not detect endogenous PLIN5 by Western Blotting using available antibodies (data not shown). Taken together, our data indicate that PEX19-mediated protein targeting to LDs is not limited to UBXD8 but includes a defined subset of LD proteins that could, potentially as a cohort, impact neutral lipid storage and consumption.

## Discussion

Our study revealed that the lack of PEX19 in cells results in a range of metabolic phenotypes, including the accumulation of very long chain fatty acids, lack of ether lipids and excess storage of neutral lipids in LDs. Importantly, we could dissect which of these phenotypes solely depend on the presence of intact peroxisomes and which are PEX19-specific and peroxisome-independent. Non-farnesylated PEX19_C296S_ is sufficient to restore fully functional peroxisomes in cells. With regard to their phospholipid and ether lipid species they are similar to wild-type cells. However, regarding neutral lipid storage and consumption, PEX19_C296S_ only partially rescued the phenotypes observed in PEX19 knock-out cells: Upon addition of exogenous oleic acid, PEX19^−/−^ cells show an anabolic defect as they produce and accumulate less TAGs than wild-type cells. This defect, however, could be fully restored in PEX19_C296S_ cells, which accumulate similar TAG amounts as wild-type cells. This suggests that peroxisome function in general is important for correct TAG synthesis under anabolic conditions.

**FIGURE 7:**
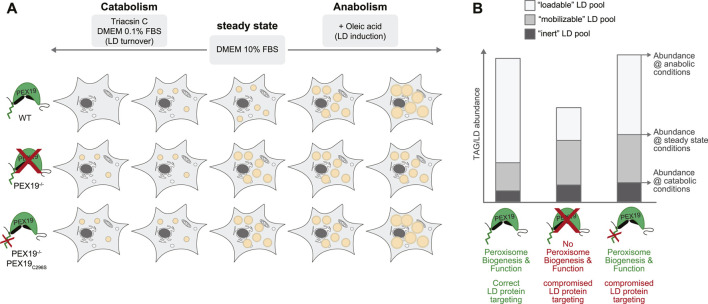
Working model for the influence of PEX19 on neutral lipid storage and consumption. **(A)** Schematic representation of the results obtained regarding TAG storage in LDs under different metabolic conditions. Abundance of LDs in wild-type (WT), PEX19^−/−^ and PEX19^−/−^PEX19_C296S_ cells under normal steady state growth conditions, under catabolic or under anabolic growth conditions is exemplified by orange circular structures. **(B)** Absolute abundance of TAG levels under catabolic growth conditions, under normal growth conditions and under anabolic conditions are indicated to scale in a stacked-bar diagram and reflect “inert” (dark grey), “mobilizable” (medium grey) and “loadable” (light grey) LD pools, respectively. Characteristics of WT, PEX19^−/−^ and PEX19^−/−^PEX19_C296S_ cells regarding peroxisome biogenesis and function as well as protein targeting to LDs are indicated. For further explanations see text.

Our data further support a model in which PEX19 affects the cell’s capacity to mobilize TAGs from LDs (Figure 7). Under normal steady state growth conditions, PEX19^−/−^ cells accumulate more TAGs and LDs than wild-type cells and importantly PEX19_C296S_ does not rescue this phenotype. This TAG/LD pool can be consumed under catabolic conditions (“mobilizable LD pool”). TAG hydrolysis *per se* therefore does not seem to be compromised in cells without wild-type PEX19, which is corroborated by our proteomics data showing that the abundance of LD-localized lipases is unchanged in PEX19^−/−^ and PEX19^−/−^PEX19_C296S_ cells. However, there is an increased pool of TAGs/LDs that seems to be non-depletable (“inert LD pool”) in these cells. Because this lipogenic phenotype persist also in PEX19_C296S_ cells, in which peroxisome function is restored, we conclude that it is independent of a general peroxisome function but specific to PEX19, presumably because of its role in ER/LD protein targeting that requires farnesylation of PEX19. Here, we have identified the PEX19-dependent LD proteome in HeLa cells, which revealed that a subset of proteins is specifically depleted from LDs when wild-type PEX19 is missing. These potential PEX19 client proteins could, as a collective, be required for correct neutral lipid storage and consumption.

The molecular mechanisms underlying LD turnover are not completely understood. While lipases such as ATGL are critical for the hydrolysis of neutral lipids that constitute the core of LDs (Zimmermann et al., 2004), it is not known which additional proteins are required to also remove the phospholipids of its encapsulating monolayer membrane and the proteins that are embedded therein. Peripheral LD proteins may be depleted from shrinking LDs by molecular crowding (Kory et al., 2015) but for some integral LD proteins it was shown that they regress to the ER membrane, probably in a process that relies on fusion of the LD with the ER (Zehmer et al., 2009). It is conceivable that complete TAG consumption and LD turnover relies on such inter-organelle contact sites. Tethering proteins that are altered in PEX19-compromised cells could directly affect such contacts. A strong candidate derived from this study is PLIN5, which mediates physical contact between LDs and mitochondria. The physiological function of PLIN5 is controversially discussed and seems to be tissue-specific and dependent on the metabolic state. While PLIN5-mediated contacts could enhance flux of fatty acids from LDs to mitochondria for enhanced beta-oxidation (Mass Sanchez et al., 2021), recent evidence suggests that LD-associated mitochondria seem to locally provide ATP to fuel local TAG synthesis (Benador et al., 2018). Further studies are required to determine, whether PEX19-dependent recruitment of PLIN5 to LDs indeed affects LD-mitochondria contacts or mitochondrial metabolism. Furthermore, spatiotemporal contacts between LDs and peroxisomes during fasting-induced lipolysis have been described (Kong et al., 2020) and it remains to be tested whether PEX19-mediated protein targeting to LDs may affect such contacts as well.

Apart from tethering molecules, physico-chemical parameters such as a certain protein/lipid ratio or lipid composition could be crucial to allow contact site formation or LD-ER fusion to deplete LDs. Loss of Rab4A in cells can lead to the accumulation of clustered LDs (Mejhert et al., 2020), which is in agreement with our observation that LDs accumulate in PEX19-compromised cells while Rab4A is depleted from LDs. Further studies are required to test whether Rab4A on LDs directly affects TAG storage or whether this is an indirect effect by for example altered glucose metabolism in cells.

In this study, we have not altered PEX19 function acutely but instead we have used cells line that stably express non-farnesylated PEX19 in a PEX19 knock-out background. Therefore, PEX19-mediated changes in subcellular protein targeting could have led to transcriptional changes in the cells, which in turn affected TAG and LD accumulation. RDH10, together with its interaction partner DHRS3, play a pivotal role in retinol metabolism. Retinoic acid modulates the transcription of numerous target genes *via* retinoic acid receptors (RAR) or peroxisome proliferator-activated receptors (PPARs) which can lead to divergent cellular responses including the regulation of oxidative capacity in mitochondria or lipogenesis (Bonet et al., 2012; Napoli, 2012). It will be interesting to see whether the subcellular localization of RDH10 affects these processes and whether PEX19-mediated recruitment of RDH10 to LDs fine-tunes the transcriptional control of lipid metabolic pathways.

PEX19 has also been implicated in the ER targeting of reticulon-homology domain containing proteins as well as in the targeting of Fis1 to mitochondria (Cichocki et al., 2018; Yamamoto and Sakisaka, 2018). It is unclear whether farnesylation of PEX19 is required for these targeting mechanisms but if these were non-peroxisomal functions of PEX19 as well, they could also contribute to the lipogenic phenotype that we observed in PEX19-compromised cells. Further organelle-specific proteomics will reveal whether PEX19-dependent alterations exist also in the ER and the mitochondrial proteome and shed light on the molecular players involved in the inter-organelle communication that ensures correct adaptation to metabolic changes.

In summary, we have shown that PEX19 plays a so far unappreciated role in the metabolic control of cells, especially under changing nutrient conditions. Importantly, we could demonstrate that the defect in correct neutral lipid storage and consumption is not simply an indirect consequence of compromised peroxisome function but that it stems from a specific defect in the peroxisome-unrelated function of PEX19 in mediating protein targeting to other lipid metabolic organelles. This study highlights that cellular metabolism relies on a sophisticated interplay of multiple organelles that have to act in concert and that shared protein targeting machinery serves as an important mechanism to dynamically adapt organelle-specific proteomes to metabolic needs.

## Methods

### Cell Culture

All cell lines used in this study have been established and described previously (Schrul and Kopito, 2016). In brief, HeLa Kyoto wild-type cells served as parental cell line to generate PEX19 knock out cells (PEX19^−/−^) using CRISPR/Cas9 technology. To generate cells that solely express non-farnesylated PEX19 (PEX19^−/−^PEX19_C296S_), PEX19^−/−^ cells were stably transfected with a plasmid encoding the PEX19_C296_ mutant. Cells were cultivated at exponential growth rates in Dulbecco´s Modified Eagle Medium (DMEM) containing 4.5 g/L glucose and pyruvate (Gibco) and supplemented with 10% FBS (Biochrom) at 37°C and 5% CO_2._ Cells were regularly tested for the absence of mycoplasma. To shift cells to catabolic conditions and to induce LD turnover, they were incubated in standard growth medium supplemented with 0.1% FBS (Biochrom) and in presence of the acyl-CoA synthetase inhibitor Triacsin C (Enzo) at a final concentration of 200 ng/ml for 18 h. To shift cells to anabolic conditions and to induce LD biogenesis, cells were treated with 200 µM oleic acid in complex with 0.2% BSA in standard growth medium for 18 h. Data reproducibility: All key findings of this manuscript with respect to the clonal PEX19-/-PEX19C296S cell line were reproducible in two different clones of this cell line (Supplementary Figure S3).

### Fluorescence Microscopy

For live-cell imaging of LDs, cells were seeded into glass bottom dishes and grown either in standard growth medium (DMEM/10% FBS) or they were shifted to catabolic conditions as outlined above. For the visualization of LDs, LipidTox Green neutral lipid dye (Life Technologies) was added to phenol-red free medium in a 1:1,000 dilution. Cells were imaged using a Zeiss Axio Observer Z1 inverted microscope equipped with a Colibri 7 LED light source, a Plan Apochromat oil objective (×63/1.4 N.A.) and appropriate filter sets. Usually, 15 z-sections with 240 nm intervals were collected using a Rolera EM-C2 camera (QImaging). Data sets were normalized for brightness/contrast, inverted and maximum intensity projections were generated Fiji version 2.1.0/1.53c.

For immunofluorescence microscopy, cells were seeded onto glass coverslips, fixed with 4% formaldehyde in PBS for 20 min, permeabilized with 0.1% Triton X-100 for 10 min, and non-specific binding sites blocked with 1% BSA in PBS. Endogenous catalase was detected using anti-catalase antibodies (Millipore cat. no. D4P7B; 1:500 dilution in 1%BSA/PBS) and Alexa Fluor 488-coupled secondary antibodies (Jackson ImmunoResearch cat. no. 711-545-152). Specimen were mounted on glass slides using Fluoromount G (EMS) and analyzed using the microscopy setup as described above.

### Shotgun-Lipidomics

Water, ethanol, and methanol in high performance liquid chromatography (HPLC) grade were acquired from Fisher Scientific (Schwerte, Germany) and all other used chemicals were purchased from Merck (Darmstadt, Germany). Lipid standards used for normalization were 06:0 PC (DHPC), 19:0 Lyso PC, 06:0 SM (d18:1/6:0), and Splash Lipidomix Mass Spec Internal Standard from Avanti Polar Lipids. For normalization of carnitine species, the standards octanoyl-L-carnitine d3 and palmitoyl-L-carnitine d3 from Supelco Analytical were used.

800.000 cells were seeded into 10 cm culture plates in standard growth medium (DMEM/10% FBS) and after 24 h shifted to anabolic or catabolic growth conditioned as outlined above and as indicated in the figure legends. Cells were grown under these conditions for additional 18 h, washed twice in cold PBS on ice and then harvested into 180 µl HPLC grade water on ice using a cell scraper. Cells were mechanically homogenized for 30 s on maximum intensity *via* Minilys (PEQLAB Biotechnologie, Erlangen, Germany). The protein concentrations of the homogenates were adjusted to 5 μg/μl using HPLC grade water after performing a bicinchoninic acid assay according to Smith et al. (1985). 100 µg protein of each sample were used for solid/liquid lipid extraction, followed by mass spectrometry analysis for the simultaneous measurement of different lipid species as described by Lauer et al. (2021). In brief, we have fixed a 96 well filter plate (0.45 µm) with circles of whatman blotting paper (6 mm diameter) placed on the wells on a 96-deep well plate (Fisher Scientific, Schwerte, Germany). A mixture of the above-described standards and 20 µl of the prepared samples were added to the whatman papers and the samples were dried under a nitrogen flow (1-2 bar) for 45–60 min. Afterwards, 20 µl of 5% PITC (v/v) diluted in ethanol/water/pyridine (1:1:1, v/v/v) were added, followed by an incubation for 20 min at room temperature. After a second drying step, lipids were extracted using 300 µl 4.93 mM ammonium acetate in methanol and shaking the plate at 450 rpm for 30 min on a plate shaker (IKA, Staufen, Germany). By centrifugation for 2 min at 500 ×g the samples were transferred into the 96-deep well plate and 600 µl 5 mM ammonium acetate in methanol/water (97:3, v/v) were used to dilute the samples. After covering the plate with a silicone mat and a further shaking step for 2 min at 450 rpm, the mass spectrometry analysis was performed on a 4000-quadropole linear-ion trap (QTRAP) equipped with a Turbo Spray ion source (AB Sciex, Darmstadt, Germany) and with help of an autosampler of Agilent HPLC 1200. The following parameters were used for lipid analysis in positive mode: measurement period = 3 min, scan type = multiple reaction monitoring (MRM), curtain gas = 20.0 psi, collision gas = medium, ion spray voltage = 5,500.0 V, temperature = 200.0°C, ion source gas 1 = 40 psi, ion source gas 2 = 50 psi, interface heater = on, entrance potential = 10 V, and collision cell exit potential = 15 V. Potential matrix effects were maximum 4.31% and in average 1.62% (see Supplementary Figure S1G).

Data and statistical analysis of Lipidomics: The Analyst 1.4.2 software from AB Sciex (Darmstadt, Germany) was used to extract the counts per second for each MRM pair and each lipid was normalized to its respective lipid class standard and the mean per technical triplicate was formed. R (R Core Team 2020; Vienna, Austria; https://www.R-project.org/) was used for statistical analysis and volcano plots were created via the R package “EnhancedVolcano” (Kevin Blighe, Sharmila Rana and Myles Lewis (2020). version 1.6.0. https://github.com/kevinblighe/EnhancedVolcano). The shown *p* value for each lipid was calculated using two-tailed student´s t-test followed by a false discovery rate correction. *p* values of processed data or ratios were calculated via ANOVA followed by tukey HSD post hoc test. Normal distribution was validated *via* qq-plot and shapiro wilk test. Homogeneity of variances was determined *via* Levene´s test. The error bars represent the standard error of the mean and statistical significance was set at *p ≤ 0.05, **p ≤ 0.01 and ***p ≤ 0.001.

### SILAC Proteomics

For stable isotope labeling, SILAC DMEM medium (Thermo) was supplemented with 10% dialized FBS, 200 μg/ml L-Proline (Sigma) and differentially isotope-labeled L-arginine (84 μg/ml) and L-lysine (146.2 μg/ml). Wild-type, Pex19^−/−^ and Pex19^−/−^PEX19_C296S_ cells were cultivated in medium containing light- (R0 and K0, Sigma), medium- (R6, K4, Cambridge Isotopes) or heavy- (R10, K8, Cambridge Isotopes) labeled amino acids, respectively, for 11 days (about 11 doubling times) with regular splitting before expansion and the induction of LD biogenesis by the addition of 200 µM oleate complexed to 0.2% BSA for 18 h.

Per cell line, 6 sub-confluent 10 cm dishes of oleate-treated cells were washed three times in PBS and then collected in ice-cold PBS using a cell scraper. Pelleted cells (4 min 500 xg at 4°C) were resuspended in 3 ml in HLM buffer [20 mM Tris/CL pH 7.5/1 mM EDTA with freshly added Complete EDTA-free protease inhibitors (Roche)] containing 250 mM sucrose. After 10 min incubation on ice, cells were mechanically lysed by passing the suspension 11 times through a ball-bearing homogenizer (Isobiotec) with 14 µm clearance. Protein concentrations of the post-nuclear supernatants (10 min, 500 ×g centrifugation at 4°C) were determined using a BCA Protein Assay Kit (Pierce). A sample from each post-nuclear supernatant was taken as an input label incorporation control (ILIC). From each cell line, post-nuclear supernatants containing 3 mg of proteins were pooled together (final total protein amount: 9 mg) and adjusted to a final concentration of 20% sucrose. The sample was transferred to a thin-walled ultracentrifugation tubes, overlaid with HLM containing 5% sucrose and with HLM without sucrose. After 1 h centrifugation at 100,000 ×g at 4°C in a SW41Ti rotor (Beckman), the buoyant LD fraction was collected using a tube slicer (Beckman). Proteins in the LD fraction were solubilized for 10 min in 2% Triton-X100 at 65°C. In parallel, proteins in the ILIC samples were solubilized in 1% SDS and 0.5% Triton X-100. Then, proteins in all samples were TCA precipitated and washed twice with ice-cold acetone. Pellets were resuspended in 0.25% Rapigest (Waters)/8 M urea/100 mM ammonium bicarbonate pH 8 by extensive vortexing and incubation at RT overnight. Proteins were reduced in 5 mM DTT for 1 h at RT and alkylated with 14 mM iodoacetamide for 45 min at RT in the dark. The urea concentration in the sample was reduced to 4 M by adding equal volumes of 100 mM ammonium bicarbonate pH 8. Peptides were digested by the addition of LysC (Promega) in an enzyme:substrate ratio of 1:50 for 4 h at RT. The urea concentration was further reduced to 1 M by the addition of 100 mM ammonium bicarbonate before tryptic (Promega) digestion with an enzyme:substrate ratio of 1:50 at RT overnight. Rapigest was removed by acidification of the trypsinized samples to pH 2 with HCL followed by sequential incubation at RT, on ice, at RT for 30 min each. Samples were cleared by centrifugation (13,000 ×g for 20 min at RT) and subjected to desalting using home-made C18 (Empore cat. no. 25515-C18) stage tips. Dried peptides were resuspended in 0.1% formic acid and subjected to mass spectrometry.

All mass spectrometry experiments were performed using an Orbitrap Fusion Tribrid mass spectrometer (Thermo Scientific, San Jose, CA, United States) with an attached Acquity M-Class UPLC (Waters Corporation, Milford, MA, United States) liquid chromatograph. A pulled-and-packed fused silica C18 with an internal diameter of 100 microns was used as a reverse phase column for analytical separations. This silica column contains 1.8 micron C18 beads (Dr. Maisch HPLC GmbH) and has a length of ∼25 cm. Separations were performed over an 80 min gradient at a flow rate of 300 nL/min. Mobile phase A consists of aqueous 0.2% formic acid and mobile phase B consists of 0.2% formic acid in acetonitrile. Peptides were directly injected onto the analytical column and the mass spectrometer was operated in a data dependent fashion with a 30 s dynamic exclusion. A Top N strategy was used, MS1 data collected in the orbitrap, and MS2 data collected in the ion trap using CID fragmentation.

Data and statistical analysis: For SILAC quantitative analysis, Proteome Discoverer 2.0 (Thermo Scientific) was used in the standard fashion to perform integration, with the Byonic v2.10.5 (Protein Metrics, Cupertino, CA, United States) node being used for protein identification assuming the SILAC heavy labeling as variable modifications. These searches were performed against a Uniprot protein database for *Homo sapiens* including isoforms concatenated with synthesized sequences. Proteolysis was assumed to be tryptic in nature, and allowed for up to two missed cleavage sites. Precursor mass accuracies were held within 12 ppm, with MS/MS fragments held to a 0.4 Da mass accuracy. Proteins were held to a false discovery rate of 1%, using standard approaches (Elias and Gygi, 2007). Label incorporation was verified to be >95% for medium and heavy isotopes.

The quantitative analysis provided three data sets, each with protein abundance signals in the wildtype control (light), *PEX19* ^*−/−*^ (medium) and *PEX19* ^*−/−*^ *PEX19*_*C296S*_ (heavy), for a total of 1,309 proteins. In an initial filtering step, 34 proteins annotated as “keratinization” or “keratin filament” in QuickGO (Binns et al., 2009) were removed as contaminants. Proteins with missing signals or that were assigned a peptide-spectrum match score <5 or unique peptides <2 by the quantitative analysis in at least one of the data sets were excluded from subsequent analyses.

For the remaining 309 proteins, the *PEX19* ^*−/−*^/wild-type and *PEX19* ^*−/−*^ *PEX19*_*C296S*_/wild-type signal ratios were log2-transformed for each data set. With SciPy 1.5.1 (Virtanen et al., 2020), the underlying data distribution was examined by fitting a normal distribution and a t-distribution to the log2-ratios. Normality was assessed and rejected by the Kolmogorov Smirnov test (*p* < 0.01), the Shapiro-Wilk test (*p* < 0.01) and the D’Agostino-Pearson test (*p* < 0.01), whereas the assumption of a t-distribution was not rejected by the Kolmogorov-Smirnov test (*p* > 0.1).

The effect of *PEX19* ^*−/−*^ and *PEX19* ^*−/−*^ *PEX19*_*C296S*_ on the proteins under investigation was identified by conducting separate two-sided paired t-tests between the log2-transformed signals of the wildtype control and *PEX19* ^*−/−*^, as well as between the control and *PEX19* ^*−/−*^ *PEX19*_*C296S*_. For *PEX19* ^*−/−*^ *PEX19*_*C296S*_/wild-type, a volcano plot of the mean log2-signal ratios and the corresponding *p*-values was generated with Matplotlib 3.2.2. The 95% confidence interval was computed based on the t-distribution of the mean log2-signal ratios with SciPy 1.5.1 (Virtanen et al., 2020). The R package pheatmap version 1.0.12 (https://CRAN.R-project.org/package=pheatmap). was used to conduct two-way hierarchical clustering of the proteins based on the relative signals of the wild-type control, *PEX19* ^*−/−*^ and *PEX19* ^*−/−*^ *PEX19*_*C296S*_.

### Western Blotting

For quantitative Western Blotting of cell lysates, cells were collected in PBS and lysed in 1% Triton X-100, 50 mM Hepes pH 7.5, 150 mM NaCl, 10% glycerol, 1 mM EDTA, 1 mM PMSF, Complete EDTA-free protease inhibitors (Roche) for 15 min on ice. Samples were cleared by centrifugation (16,000 ×g, 10 min, 4°C) and the protein concentration determined using a BCA assay (Pierce). Equal protein amounts were separated by SDS–PAGE followed by wet-transfer onto nitrocellulose membranes.

For assessing the quality of the LD fractionation process, samples taken before (PNS) or during the fractionation procedure were analyzed by SDS-PAGE followed by wet-transfer onto nitrocellulose membranes. Nonspecific binding was blocked with 5% skimmed milk in TBS-T. Primary antibodies (anti-PEX19: ab137072, Abcam; 1:1,000; anti-UBXD8: 16251-1-AP, PTG; 1:1,000; anti-Tubulin: T6199, Sigma-Aldrich; 1:10,000; anti-Calnexin: ADI-SPA-865, Enzo Life Sciences; 1:2,000 anti-RDH10: 14644-1-AP, PTG; 1:1,000; anti-ATGL: 2138, Cell Signaling; 1:2,000; anti-Rab4A: 610,888, BD Biosciences; 1:1,000) were diluted in the same blocking solution. IRDye secondary antibodies were purchased from LiCor (926-68020, 926-32211) used in a 1:15,000 dilution in blocking solution and used for signal detection by the Odyssey CLx infrared imaging system (Licor). For western blots detecting Rab4A in LD fractions, we used the anti-Rab4A and anti-mouse HRP-coupled (115-035-003, Dianova; 1:20,000) antibodies and detected the signals by enhanced chemiluminescence (Thermo Scientific Super Signal West Femto) and the Amersham Imager 600 RGB imaging system. For quantification, band intensities were quantified by densitometry using the Image Studio Lite Software (LiCor) and graphs were generated using GraphPad Prism Software.

## Data Availability

The original contributions presented in the study are included in the article/Supplementary Material, further inquiries can be directed to the corresponding author. The mass spectrometry proteomics data in the study have been deposited to the ProteomeXchange Consortium via the PRIDE (Perez-Riverol et al., 2022) partner repository with the dataset identifier PXD032200.

## Supplementary Material

Supplementary Table 1 Excel file with lipidomics data.

Supplementary Table 2 Excel file with proteomics data.
