# Supplementary material for: PEX19 Coordinates Neutral Lipid Storage in Cells in a Peroxisome-Independent Fashion
Source: Front Cell Dev Biol. 2022 Apr 26;10:859052. doi: 10.3389/fcell.2022.859052 (PMC9086359; doi:10.3389/fcell.2022.859052)
Supplement: Supplementary file 2 [file DataSheet1.pdf]

Supplementary Figures S1-S3

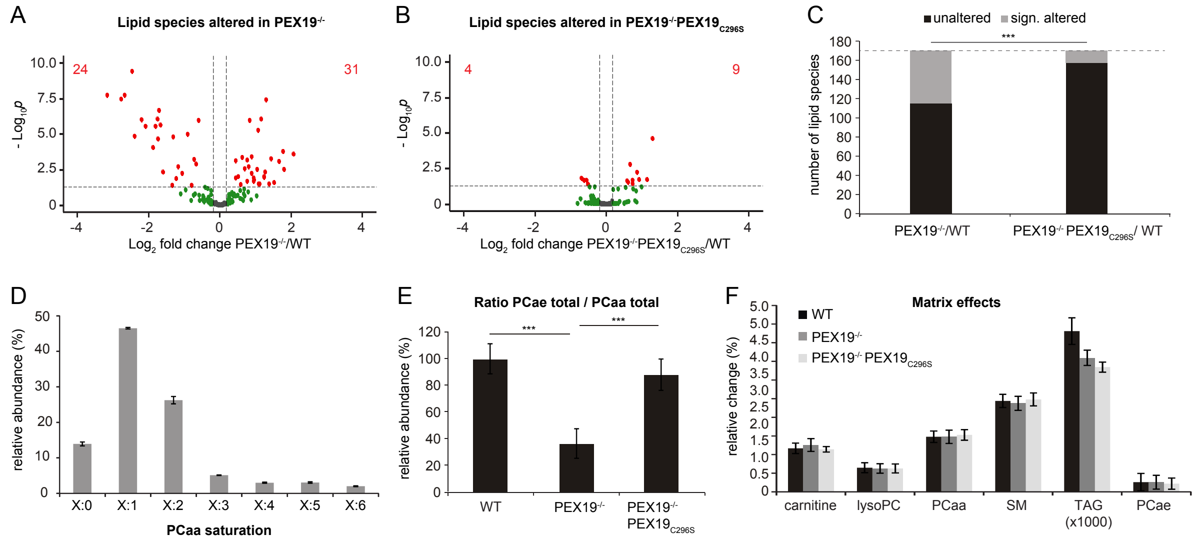

Lyschik *et al.*, Figure S1

Figure S1 (related to Figure 2): Stable expression of non-farnesylated PEX19 rescues many, but not all lipid metabolic alterations in PEX19$^{-/-}$ cells.

(A+B) Volcano plots indicate fold changes of 170 distinct lipid species analyzed by shotgun-lipidomics that are logarithmically plotted against the p-value (derived from unpaired, two-sided student´s t-test) after FDR-correction, illustrating changes in PEX19$^{-/-}$ cells **(A)** and PEX19$^{-/-}$PEX19$_{C296S}$ cells **(B)** compared to WT cells, respectively. Grey dots: lipid species without significant changes. Green dots: lipid species with a fold change greater than the average SEM but with a p-value >0.05. Red dots: significantly altered (p-value <0.05) lipid species with a greater fold change than the average SEM. **(C)** Bar graph showing the number of unaltered and significantly altered lipid species in PEX19$^{-/-}$ cells and PEX19$^{-/-}$PEX19$_{C296S}$ cells compared to WT cells, respectively. Chi-squared test analyses reveal that the lipidome in PEX19$^{-/-}$ and PEX19$^{-/-}$PEX19$_{C296S}$ cells is significantly distinct with a p-value <0.00001. **(D)** Bar graph showing the relative abundance of PCaa species with distinct acyl chain saturation as a fraction of total PCaa in WT cells. **(E)** Bar graph showing the relative abundance of the ratio of total PCae/ total PCaa in WT, PEX19$^{-/-}$ and PEX19$^{-/-}$PEX19$_{C296S}$ cells. Error bars indicate SEM and statistical significance between the groups was determined by an ANOVA followed by Tukey post-hoc test. *** indicates a p-value <0.001. Non-significant changes are not indicated. For each cell line 11 individual samples were analyzed with three technical replicates each. **(F)** Matrix effects**:** The calculated matrix effects of the measured parameters carnitine, lyso-phosphatidylcholines (lyso PC), phosphatidylcholines (PCaa), sphingomyelins (SM), triacylglycerides (TAG) and phosphatidylcholines plasmalogens (PCae) are shown in a bar chart.

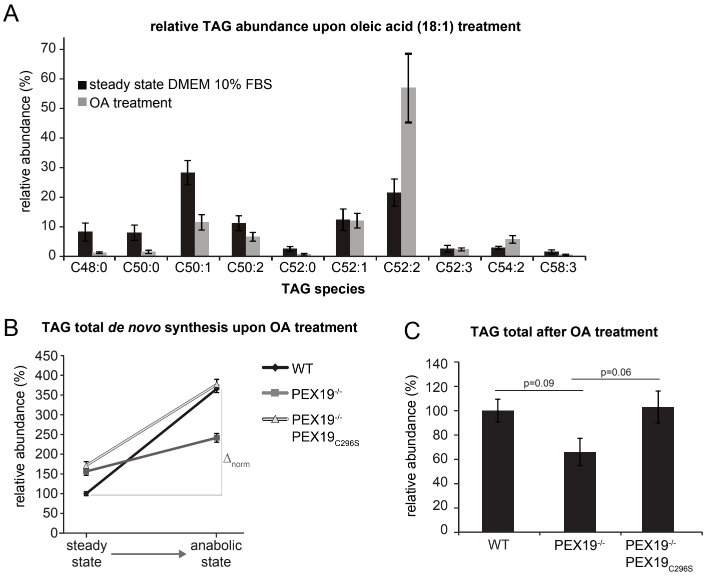

Lyschik *et al.*, Figure S2

Figure S2 (related to Figure 4): *De novo* TAG synthesis is not compromised in $PEX19^{-/-}PEX19_{C296S}$ cells.
(A) Exogenous oleic acid (OA) is predominantly incorporated into TAG C52:2 species. Shotgun-lipidomics results for TAG species as indicated in WT cells before and after oleic acid treatment. **(B+C)** Shotgun-lipidomics results for all TAG species (10 species in total) upon OA treatment. B: Plot indicating *de novo* synthesis of all TAG species upon OA treatment for WT, $PEX19^{-/-}$ and $PEX19^{-/-}PEX19_{C296S}$ cells (Starting points = steady state; endpoints = anabolic state). Values were normalized to 100% in WT cells under steady state conditions before OA treatment. Relative TAG *de novo* synthesis as fold change is defined by the ratio between C52:2 levels under anabolic condition and C52:2 levels at steady state before OA treatment and is 3.7 for WT, 1.6 for $PEX19^{-/-}$ and 2.2 for $PEX19^{-/-}PEX19_{C296S}$ cells. Δ_{norm} indicates the absolute TAG synthesis as defined by the difference of TAG levels under anabolic conditions and TAG levels at steady state before OA treatment and is 266 for WT, 85 for $PEX19^{-/-}$ and 205 for $PEX19^{-/-}PEX19_{C296S}$ cells. **(C)** Bar diagram showing the relative abundance of all TAG species accumulating after OA treatment in WT, $PEX19^{-/-}$ and $PEX19^{-/-}PEX19_{C296S}$ cells. Values were normalized to 100% in WT cells. Error bars indicate SEM.

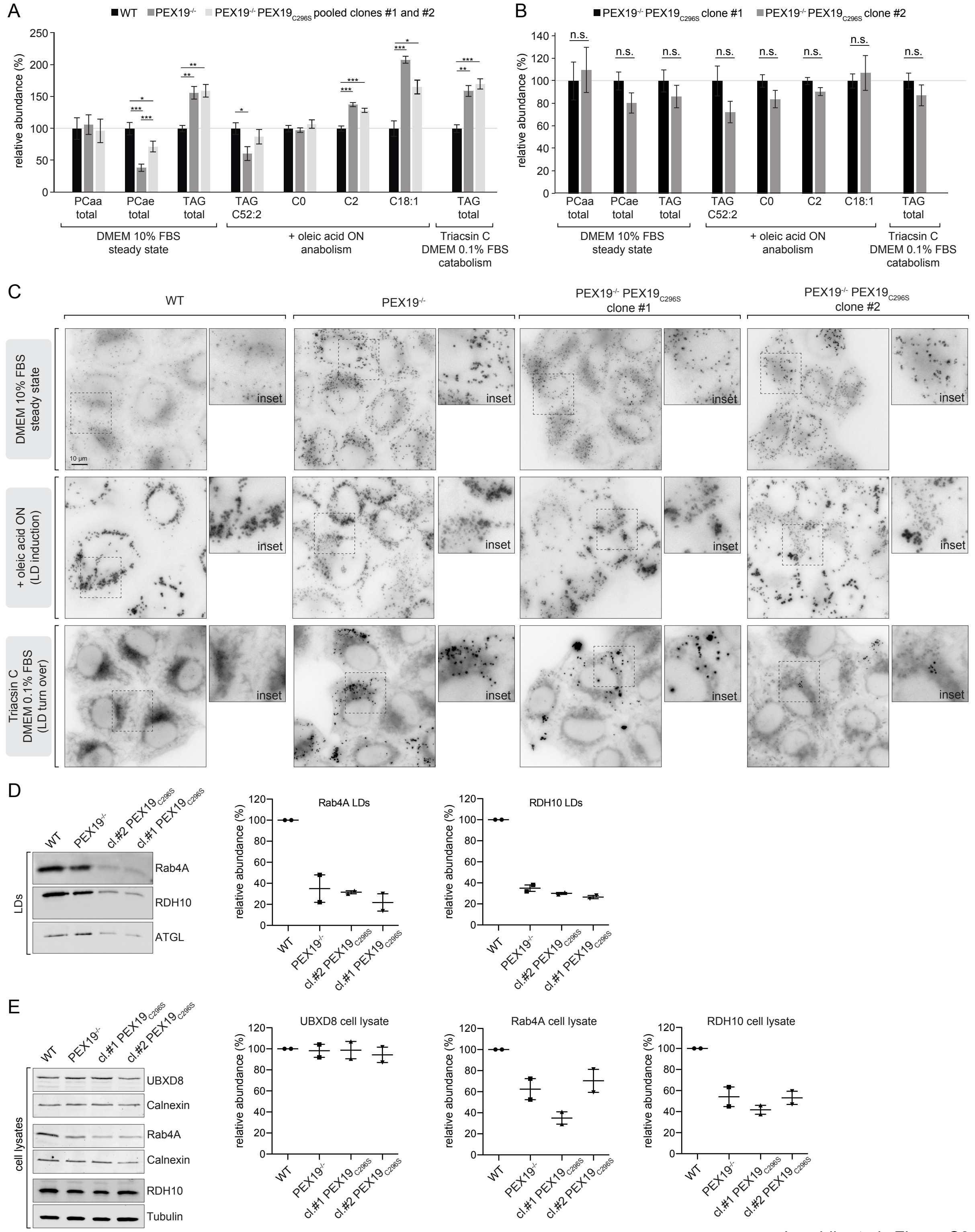

Lyschik *et al.*, Figure S3

Figure S3: Comparison of two different clones of the PEX19$^{-/-}$PEX19$_{C296S}$ cell line (A) Key findings of lipidomic analyses are recapitulated when pooled data of two different clones of the PEX19$^{-/-}$PEX19$_{C296S}$ cell line were compared to those of WT and PEX19$^{-/-}$ cells. The bar diagram shows shotgun-lipidomics results for various lipid species under different metabolic conditions as indicated. Error bars indicate SEM and statistical significance between the groups was determined by an ANOVA followed by

Tukey post-hoc test. *, **, *** indicate p-values <0.05, <0.01, and <0.001, respectively. Non-significant changes are not indicated. Compare graphs to data in Figures 1A, 1F, 3A, 4C, 4D, 5C. **(B)** Two different clones of the PEX19$^{-/-}$PEX19$_{C296S}$ cell line are indistinguishable from each other with respect to the key findings of the lipidomics analyses. The bar diagram shows shotgun-lipidomics results for various lipid species under different metabolic conditions as indicated. Error bars indicate SEM and statistical significance between the two groups was determined by a two-tailed, two-way student´s t-test. Non-significant changes are indicated as n.s.. **(C)** Side-by-side comparison of LD staining under varying metabolic conditions. Micrographs show LipidTox-staining of living WT and PEX19$^{-/-}$ cells as well as of two different clones of the PEX19$^{-/-}$PEX19$_{C296S}$ cell line as indicated. Inverted, maximum intensity projections of z-stacks are shown. Scale bar: 10 µm. Please note that the micrographs of WT, PEX19$^{-/-}$ and PEX19$^{-/-}$PEX19$_{C296S}$ clone #1 cells under “steady state” conditions and upon Triacsin C / 0.1% FBS treatment are also shown in the main manuscript in figures 1C and 5H, respectively, and are here shown again for direct comparison of LipidTox-staining upon different treatments and for comparison with the second clone of the PEX19$^{-/-}$PEX19$_{C296S}$ cell line. **(D)** Validation of PEX19-dependent LD proteins by Western Blotting. Left: Representative Western blots of LD fractions from WT, PEX19$^{-/-}$ and two different clones of the PEX19$^{-/-}$PEX19$_{C296S}$ cell line (PEX19$_{C296S}$ cl.#1 and cl.#2) using antibodies as indicated. ATGL served as loading control. Right: Scatter plots indicate the relative protein abundance of proteins in LD fractions derived from Western Blots as shown on the left. Signals were quantified by densitometry and relative abundances were calculated as ratio of the signals of interest to the corresponding ATGL signal in the same lane. Values derived from two independent Western blots are shown and were normalized against the values from WT cells, which were set to 100%. **(E)** Left: Western Blot analyses of cell lysates from WT, PEX19$^{-/-}$ and two different clones of the PEX19$^{-/-}$PEX19$_{C296S}$ cell line (PEX19$_{C296S}$ cl.#1 and cl.#2) using antibodies as indicated. Anti-tubulin and anti-calnexin signals served as loading controls, respectively, and were used to normalize anti-UBXD8, Rab4A and RDH10 signals. Right: Scatter plots indicate the relative protein abundance of proteins in cell lysates derived from Western Blots as shown on the left. Signals were quantified by densitometry and relative abundances were calculated as ratio of the signals of interest to the corresponding loading control (either calnexin or tubulin) in the same lane. Values derived from two independent Western blots are shown and were normalized against the values from WT cells, which were set to 100%.
